# Defects Engineering with Multiple Dimensions in Thermoelectric Materials

**DOI:** 10.34133/2020/9652749

**Published:** 2020-05-22

**Authors:** Chenxi Zhao, Zhou Li, Tianjiao Fan, Chong Xiao, Yi Xie

**Affiliations:** ^1^Hefei National Laboratory for Physical Sciences at Microscale, CAS Center for Excellence in Nanoscience, University of Science & Technology of China, Hefei, Anhui 230026, China; ^2^Institute of Energy, Hefei Comprehensive National Science Center, Hefei, Anhui 230031, China

## Abstract

Going through decades of development, great progress in both theory and experiment has been achieved in thermoelectric materials. With the growing enhancement in thermoelectric performance, it is also companied with the complexation of defects induced in the materials. 0D point defects, 1D linear defects, 2D planar defects, and 3D bulk defects have all been induced in thermoelectric materials for the optimization of thermoelectric performance. Considering the distinct characteristics of each type of defects, in-depth understanding of their roles in the thermoelectric transport process is of vital importance. In this paper, we classify and summarize the defect-related physical effects on both band structure and transport behavior of carriers and phonons when inducing different types of defects. Recent achievements in experimental characterization and theoretical simulation of defects are also summarized for accurately determining the type of defects serving for the design of thermoelectric materials. Finally, based on the current theoretical and experimental achievements, strategies engaged with multiple dimensional defects are reviewed for thermoelectric performance optimization.

## 1. Introduction

Thermoelectric technology, catering for the growing demand for sustainable energy and special devices, has been in continuous development for decades of years. Achieving direct conversion between thermal and electrical energy without emission or any moving parts [1–3], the unique advantage of TE equipment enables the broad prospects for the future research and application. However, even though there is no limitation in the energy conversion efficiency of the TE equipment, the TE equipment is not advanced enough owing to the limitation in thermoelectric materials. The energy conversion efficiency is positively correlated with the dimensionless figure of merit *ZT*, which qualifies the performance of TE materials and is defined as *ZT* = *σS*^2^*T*/*κ*, where *σ* is the electrical conductivity, *S* is the Seebeck coefficient, *T* is the absolute temperature, and *κ* is the thermal conductivity equal to the sum of carrier thermal conductivity (*κ*_*e*_) and lattice thermal conductivity (*κ*_*L*_) [4–6].Apparently, thermoelectric materials with high performance demand large electrical conductivity, large Seebeck coefficient, and low thermal conductivity. However, these physical parameters are not independent but couple with each other. The manipulation of a single physical parameter is bound to influence other parameters and always shows an undesirable trend, such as the opposite trend of electrical conductivity and Seebeck coefficient as a function of carrier concentration [7, 8]. The coupling characteristics of thermoelectric parameters set an obstacle for the optimization of thermoelectric performance, which also pushes us into clarifying the physical effects when inducing defects in the thermoelectric materials.

To achieve advanced thermoelectric materials with high performance, on the one hand, it is essential to search for materials with favorable intrinsic properties such as high band degeneracy or lattice anharmonicity [2, 9, 10], while, generally, undoped ingots seldom show an ideal thermoelectric performance. Hence, it is also of vital importance to realize the optimization of thermoelectric performance. Fortunately, defects in the materials provide a new degree of freedom for tuning thermoelectric transport behavior, which expands the space for tuning the physical parameters to optimize thermoelectric performance [8]. In fact, most of the advanced thermoelectric materials are doped materials, which emphasize the significance of 0D point defects in thermoelectric materials. Benefited from the thermoelectric transport theories and concepts, now we have recognized that point defects have an impact on both band structure and transport behavior of carriers and phonons [8, 11–13]. However, for the diversity and sensibility to the composition and the preparation process, accurate characterization and theoretical simulation to judge the type and distribution of point defects in the material should also be taken seriously. Benefited from the development of material synthesis and processing technique, nanostructures can be already constituted in the thermoelectric materials which also induce large amounts of 2D planar defects and 3D bulk defects [14–16]. The construction of heterojunction between the matrix and inclusions can tune the electrical transport process. And the differences between the mean free paths of phonons and electrons enable the reduction in *κ*_*L*_ while not excessively deteriorating the electrical properties [13, 15, 17, 18]. Recently, 1D dislocation with high concentration has also been found and induced in thermoelectric materials exhibiting a remarkable virtue of strongly reducing *κ*_*L*_ due to the unique middle-frequency phonon scattering [19–22]. It is worthwhile to note that materials designed with 1D~3D defects are often intentionally accompanied with 0D point defects, or in other words, materials with 0D point defects are chosen as the matrix [18–28]. The engagement of different types of defects realizes further enhancement of thermoelectric performance, and it also complicates and diversifies the way to optimize the properties of thermoelectric materials.

With the complication of defects induced in the materials, understanding the role of each kind of defects in depth is of significant importance. In this paper, we classify and summarize the defect-related physical effects with different dimensions regarding both influences on carrier and phonon transport behaviors. Characterizations and theoretical simulation of defects are also summarized for accurately determining the type of defects in the materials. Faced on the process of optimizing thermoelectric performance for a certain system via manipulating defects, we hope the work could help the interested readers have a better design of thermoelectric materials when considering inducing defects.

## 2. Point Defects

**Figure 1:**
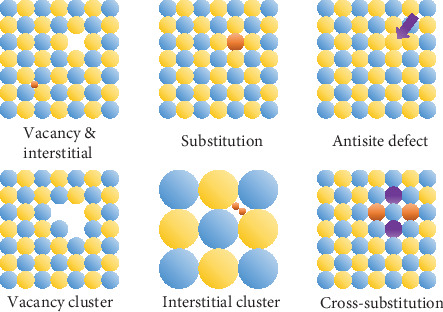
Schematic diagram of different kinds of point defects.

Point defects are zero-dimensional defects at or around which the atomic arrangement deviates from the normal crystal lattice. Typically, point defects contain vacancies, interstitial atoms, and substitution atoms, and additionally, complex defects occurring in several crystal lattice sites such as vacancy clusters, interstitial clusters, and cross-substitution can also be generally regarded as point defects [29–31]. Point defects can be spontaneous, such as anion vacancies generated due to the relative low enthalpy of vaporization. Point defects can also be introduced according to the wishes of the researchers, among which the most common and easy to achieve can be elemental doping.  Figure 1 shows the schematic diagram of various types of point defects including common simple point defects and complex point defects. From an intuitive point of view, the point defects cause lattice distortion, around which the periodic potential field is deviated from the normal condition, and consequently become the scatter source of electrons or phonons. From another intuitive point of view, the point defects cause charge imbalance, inducing extrinsic carriers which change the electric transport properties. As for isoelectronic doping, although the chemical valence seems to be in balance, owing to the difference of electronegativity and atomic radius, the dopants can form an isoelectron trap [32–36] or alter the dominance of distinct types of point defects, both of which have an impact on the electrical properties. From the perspective of reciprocal lattice, the defects change the electronic and phonon structures which determine the intrinsic electrical and thermal transport properties. Obviously, it is of vital importance to bridge a relationship between the defects and thermoelectric transport properties, and to clarify how the point defects affect the electron and phonon transport process. Here, we summarize various types of point defects, as well as direct or indirect characterization methods to afford proof for the existence of the specific point defects. We also sum up the physical effects induced from the point defects and their meaning for optimization of thermoelectric properties.

Most intuitively, point defects break the lattice symmetry, distorting the periodic potential field, which changes the trajectory of transport electrons. Supposed there is no energy degradation during the carrier scattering process, the carrier mobility *μ*_*c*_ can be expressed as [37]

(1) $$\begin{array}{c} \mu_{c}=\frac{e\tau_{c}}{m^{*}}, \end{array}$$

where *τ*_*c*_ is the average relaxation time and *m*^∗^ is the effective mass. Here, we can clearly see that the carrier mobility can be regulated via two ways: regulate the *m*^∗^ related to the band structure and regulate *τ*_*c*_ related to the carrier scattering effects. In materials with specific chemical composition, temperature and defects will both affect the scattering mechanism of materials, contributing to different impacts on the electrical properties and carrier thermal conductivity.

Therefore, it is significant to judge which scattering mechanism dominates a certain temperature region and to have an in-depth understanding about the influence of defects on the scattering mechanisms. Generally, with the increasing temperature, the dominance of the carrier scattering mechanism will be altered in semiconductors. Commonly, around room temperature or particularly at low temperature where phonon effects are limited [38], the ionization scattering originated from dopant atoms or intrinsic point defects occupies the dominant position, and the dependence among carrier mobility, temperature, and ionized impurity concentration is regarded as [39]

(2) $$\begin{array}{c} \mu_{c,\text{ion}}\propto \frac{T^{3/2}}{N_{I}}, \end{array}$$

where *N*_*I*_ is the total ionized impurity concentration and equal to the sum of donor impurity concentration and acceptor impurity concentration. This tells us that a large dopant concentration will reduce carrier mobility, and with the increasing temperature, because the increased carrier random thermal velocity reduces the period of carriers moving around the additional potential field induced from ionized impurities, the carrier mobility will increase and the ionization scattering will be weakened. In the case of ionization scattering, according to the Brooks-Herring formula [38], under Dingle and Brooks-Herring approximation, the carrier relaxation time is expressed as

(3) $$\begin{array}{c} \tau_{c,\text{ion}}=\frac{16\sqrt{2}{\pi \varepsilon }^{2}{\left(m^{*}\right)}^{1/2}E^{3/2}}{N_{I}Z^{2}e^{4}}{\left[\ln\left(1+b\right)-\frac{b}{1+b}\right]}^{-1}, \end{array}$$

(4) $$\begin{array}{c} b=\frac{8m^{*}E}{\hbar^{2}{\beta_{S}}^{2}}, \end{array}$$

where *ε* is the static permittivity of the material, *N*_*I*_ is the ionized impurity concentration, *Z* is the charge on the impurity in units of *e*, *E* is the energy of an electron, *ℏ* is reduced Planck's constant, and *β*_*S*_ is the inverse screen length. Under the Born approximation which presupposes that *b* ≫ 1, the quantity within the bracket in Equation (3) can be simplified as ln*b* for nondegenerate semiconductors, and Equations (3) and (4) can also be further converted into *T*-dependent engaging with *E* = 3*k*_*B*_*T*, and Brooks-Herring (BH) mobility can be obtained [38]. According to Equations (3) and (4), we can find that except for the intrinsic material parameters such as *ε*, *τ*_*c*_ shows a strong relationship with the parameters related to point defects such as *N*_*I*_ and *Z*, which indicates that large defect concentration and large charge on the impurity lead to the decrease in carrier relaxation time and the intension in the ionized impurity scattering.

When the temperature is further increased, particularly in middle- or high-temperature regions, the defect-related ionized impurity scattering is gradually weakened, but acoustic phonon scattering gradually dominates. In a nondegenerate case and for acoustical vibrations of the lattice, the dependence between carrier mobility and temperature can be given as [37]

(5) $$\begin{array}{c} \mu_{c,\text{acoustic}}\propto T^{-3/2}. \end{array}$$

Obviously, the carrier mobility decreases with the increasing temperature due to the intensified interaction between lattice vibration and moving carriers. Besides, for the materials with low defect concentration, the ionized impurity concentration is circumscribed but acoustic phonon scattering can dominate at a low temperature.

When the temperature exceeds the Debye temperature *θ*_*l*_ (*θ*_*l*_ = *ℏω*_*l*/_*k*_0_), polar scattering by the optical vibration dominates, and the dependence between carrier mobility and temperature can be given as [37]

(6) $$\begin{array}{c} \mu_{c,\text{optical}}\propto T^{-1/2}, \end{array}$$

according to which the carrier mobility also decreases with the increasing temperature, and the lattice vibration dominates while the impurity scattering is limited. Besides, it should be noted again that the formulas mentioned above are based on simple elastic carrier scattering, while in the case of inelastic carrier scattering, the formulas may be inapplicable [37, 39].

**Figure 2:**
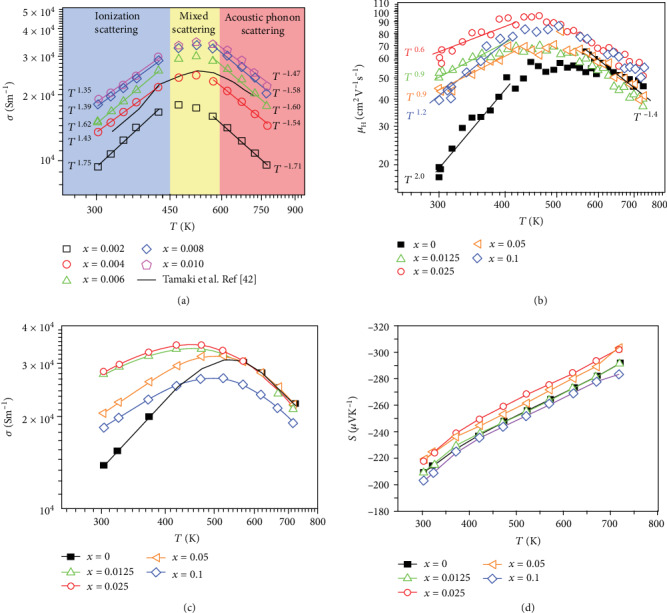
Carrier scattering mechanism and related electrical properties. (a) Temperature-dependent electrical conductivity of Mg_3.2_Sb_1.5_Bi_0.5-__*x*_Te_*x*_ [40, 42]. Reproduced with permission from RSC Publishing. (b) Hall mobility, (c) electrical conductivity, and (d) Seebeck coefficient of Mg_3.2-__*x*_Mn_*x*_Sb_1.5_Bi_0.49_Te_0.01_ as functions of temperature [41]. Reproduced with permission from Elsevier Ltd.

However, all of the above are ideal states in which a certain scattering mechanism dominates. In fact, mixed scattering mechanisms in which two scattering mechanisms work together also exist, especially in the temperature range that the scattering transforms from one scattering mechanism to another [40, 41]. As shown in Figure 2(a), Shuai et al. [40, 42] considered that in the Mg_3.2_Sb_1.5_Bi_0.5−*x*_Te_*x*_ system, when bipolar conduction does not occur, *n*_*H*_ is almost a constant, so the temperature dependence of electrical conductivity and Hall mobility can be seen as the same. In this case, the electrical conductivity *σ* ∝ *T*^3/2^ below 450 K corresponds to ionized impurity scattering, and *σ* ∝ *T*^−3/2^ above 600 K corresponds to acoustic phonon scattering, but if between 450 K and 600 K, there is no obvious specific functional relationship. This means a mixed scattering mechanism exists in which ionized impurity scattering and acoustic phonon scattering works together. A similar result can also be found in the work of Chen et al. [41], in which as for Mg_3.2_Sb_1.5_Bi_0.49_Te_0.01_, the Hall carrier mobility *μ*_*H*_ ∝ *T*^2^ below 400 K, *μ*_*H*_ ∝ *T*^−1.4^ above 600 K, and between 400 K and 600 K, have no specific temperature dependence, which means the existence of a mixed scattering mechanism.

In a common sense, the more point defect concentration, the less carrier mobility according to Equations (2)–(4) for the intensified ionized impurity scattering. However, Shuai et al. found that in the Mg_3_(Sb,Bi)_2_ system doping concentration and carrier mobility have the same trends via Nb doping [40]. Afterwards, Chen et al. also found a similar result via Mn doping in the Mg_3_(Sb,Bi)_2_ system. As shown in Figure 2(b), an abnormal trend can be found that Hall mobility increases with the increasing doping concentration at a low temperature. The abnormal trend can be explained that the dopant atoms changed the scattering mechanism. Mg_3.2−*x*_Sb_1.5_Bi_0.49_Te_0.01_ without Mn doping shows a trend that *μ* ∝ *T*^2.0^. However, after Mn doping, the Hall mobility is increased and shows a temperature dependence corresponding to mixed scattering mechanism. The results illuminate that Mn doping changed the carrier scattering mechanism from ionized impurity scattering to mixed scattering, hence realizing an abnormal enhancement in Hall mobility and electrical conductivity (shown in Figure 2(c)). Besides, as shown in Figure 2(d), coupled with multiple conduction bands, the Seebeck coefficient is enhanced, which means the achievement in decoupling enhancement in both electrical conductivity and Seebeck coefficient below 500 K, which also devotes to a large enhancement in power factor and *ZT* value [41]. The strategies of altering carrier scattering via inducing point defects undoubtedly provide a new idea for optimizing thermoelectric performance.

**Figure 3:**
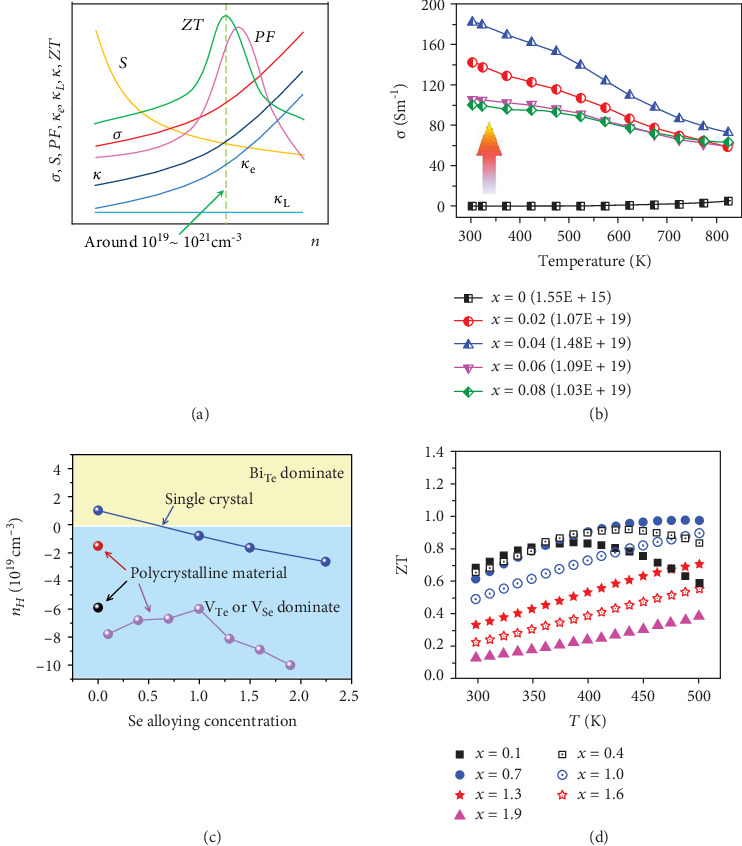
(a) Schematic diagram showing characteristics of carrier concentration dependence of thermoelectric-related parameters. (b) Carrier concentration at room temperature (shown in brackets) and temperature-dependent electrical conductivity of Bi_2−*x*_La_*x*_O_2_Se. The increased carrier concentration benefited from doping atoms leads to a significant enhancement in electrical conductivity [33]. Reproduced with permission from the American Ceramic Society. (c) Room temperature carrier concentration as a function of alloying concentration in the Bi_2_Te_3−*x*_Se_*x*_ series. The yellow region represents Bi_Te_ is the dominating defects while the blue region representing V_Te_ or V_Se_ is the dominating defects. Data are from [56, 58, 62]. (d) Temperature dependence *ZT* value of polycrystalline Bi_2_Te_3−*x*_Se_*x*_ series prepared via hot deformation [56]. Reproduced with permission from WILEY-VCH.

Another significant feature of point defect is causing charge imbalance which changes the carrier concentration of the material, which has been an effective and widely utilized strategy for optimizing thermoelectric performance. Generally, for the coupling characteristics of thermoelectric parameters, the optimized carrier concentration is located at around 10^19^~ 10^21^ cm^−3^ as shown in Figure 3(a). Pristine ingots seldom exhibit ideal thermoelectric performance, but fortunately, point defects provide a feasible way to tune the carrier concentration to the optimized region. As for the materials with low intrinsic electrical conductivity such as BiCuSeO and Bi_2_O_2_Se, aliovalent doping (including inducing vacancies or interstitial atoms) has been successfully used to induce excessive carriers and enlarge the carrier concentration, further increasing the electrical conductivity to realize the enhancement in *ZT* [43–46]. In contrast, as for the materials with extortionate carrier concentration, aliovalent doping can also be utilized to compensate the carrier concentration to limit the electronic thermal conductivity and enhance the Seebeck coefficient [47–49]. Besides, although isoelectronic doping does not directly lead to imbalances in chemical valence, the difference in charge density and electronegativity may also lead to the changes in carrier concentration or even the type of dominant carriers. Isoelectronic trap is a kind of carrier bound state originating from isoelectronic doping. The difference of electronegativity between dopant atoms and pristine atoms means the difference of the ability to attracting carriers, which generates a carrier bound state around the isoelectronic dopant atom. For the low electronegativity dopant atoms, the ability to attract holes is stronger, which facilitates the formation of a hole bound state, triggering the excitation of electrons and increasing the concentration of electrons. In contrast, the high electronegativity dopant atoms will form an electron bound state which increases the concentration of holes [32, 33]. The introduction of isoelectronic traps has been successfully utilized to optimize carrier concentration in La-doped Bi_2_O_2_Se, In-doped Sr_8_Ga_16_Ge_30_, Mn-doped YbZnSb_2_, and S-doped MnTe [32–36]. Figure 3(b) shows the temperature dependence of electrical conductivity and the carrier concentration at room temperature in La-doped Bi_2_O_2_Se materials. The electronegativity of La (1.10) is much lower than that of Bi (2.02); hence, the La dopant atoms can form the hole bound state and increase the carrier concentration for n-type Bi_2_O_2_Se. As shown in Figure 3(b), the carrier concentration of pristine Bi_2_O_2_Se (shown in brackets) is only 1.55 × 10^15^ cm^−3^ indicating the characteristics of insulators. After La doping, the carrier concentration is boosted up to ~10^19^ cm^−3^ corresponding to the characteristic of a degenerate semiconductor, which contributes to the significant enhancement in electrical conductivity from 0.03 Scm^−1^ to 182 Scm^−1^. Profited from the boosted electrical conductivity, the *ZT* value of Bi_2−*x*_La_*x*_O_2_Se is significantly enhanced, which shows the feasibility of inducing isoelectronic trap to optimize carrier concentration and *ZT* value [33].

**Figure 4:**
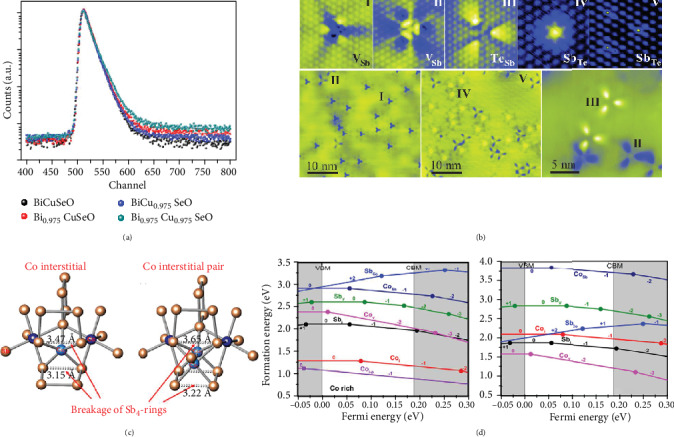
Characterizations for point defects and calculations for complex point defects. (a) Positron lifetime spectrum of Bi_1-__*x*_Cu_1-__*y*_SeO providing evidence for the existence of V_Bi_ and V_Cu_ [84]. Reproduced with permission from the American Chemical Society. (b) STM images of Sb_2_Te_3_ providing evidence for the existence of various kinds of defects [52]. Reproduced with permission from the American Physical Society. (c) Crystal structure of CoSb_3_ with single Co interstitial and Co interstitial pair, respectively [29]. (d) Calculated defect formation energy as a function of *E*_F_ in Co-rich and Sb-rich regions, respectively, providing evidence for the existence of Co interstitial pair complex defect at low temperature [29]. Reproduced with permission from the American Chemical Society.

Except for the isoelectronic trap effect, the isoelectronic doping can also tune the carrier concentration through the approach that extrinsic dopant tunes the native defects. The (Bi,Sb)_2_(Te,Se)_3_ system is a typical instance for the case. The pristine Bi_2_Te_3_ single crystal always shows a p-type conduction mechanism, which originated from the intrinsic Bi_Te_′ antisite defects [50, 51].  Figure 1 shows the schematic diagram of the antisite defects, which means one host atom occupies the position of another host atom. The antisite defects in Sb_2_Te_3_- and Bi_2_Te_3_-based solid solution have been observed by scanning tunneling microscopy (STM) as shown in Figure 4(b) [52, 53]. As for stoichiometric Bi_2_Te_3_, because the enthalpy of vaporization and boiling point of Bi (151 kJ/mol, 1837 K) is much higher than that of Te (114.1 kJ/mol, 1261 K) [54], Te is easier to vaporize, causing the composition of the products to be usually rich in bismuth and producing a large number of V_Te_^••^ which can be occupied by cations. It has been pointed out that the smaller difference in bond polarity (or it can also be explained that the smaller difference in electronegativity), the less resistance against forming antisite defects [50]. Because the electronegativity of Bi (1.9) is similar to that of Te (2.1) [54], Bi is easy to occupy with V_Te_^••^-forming Bi_Te_′ antisite defects. The formation of Bi_Te_′ antisite defects can be described by the following defect equation [55]:

(7) $$\begin{array}{c} {\text{Bi}}_{2}{\text{Te}}_{3}⇌\left(2-\frac{2}{5}x\right){\text{Bi}}_{\text{Bi}}^{\times }+\left(3-x\right){\text{Te}}_{\text{Te}}^{\times }+x\text{Te}\left(g\right)↑+{\left(\frac{2}{5}x{\text{V}}_{\text{Bi}}^{\prime \prime \prime }+\frac{3}{5}x{\text{V}}_{\text{Te}}^{••}\right)}^{\times }+\frac{2}{5}x\text{B}{\text{i}}_{\text{Te}}^{\prime }+\frac{2}{5}h^{•}, \end{array}$$

From the defect equation, it is obvious that the formation of Bi_Te_′ antisite defects produces excessive holes making the Bi_2_Te_3_ single crystal perform a p-type conductivity. However, the polycrystalline stoichiometric Bi_2_Te_3_ shows an n-type conductivity mechanism as shown in Figure 3(c) [56], which can be attributed to the changes of dominant defects during the plastic deformation process. It has been reported that p-type Bi_2_Te_3_ casting ingots transform into n-type after heavy plastic deformation by extrusion [57]. Zhao et al. also reported that the pristine Bi_2_Te_3_ prepared via ball milling shows an n-type conductivity mechanism [58]. The impact of plastic deformation on the conductivity can be explained by the donor-like effect, an interaction of vacancies with antisite defects during the processing shown as follows [58, 59]:

(8) $$\begin{array}{c} 2{\text{V}}_{\text{Bi}}^{\prime \prime \prime }+3{\text{V}}_{\text{Te}}^{••}+\text{B}{\text{i}}_{\text{Te}}^{\prime }⇌{\text{V}}_{\text{Bi}}^{\prime \prime \prime }+{\text{Bi}}_{\text{Bi}}^{\times }+4{\text{V}}_{\text{Te}}^{••}+6e^{\prime }. \end{array}$$

This means that plastic deformation can enhance the electron concentration, and hence, polycrystalline stoichiometric Bi_2_Te_3_ is an n-type semiconductor. Except for the donor-like effect, Liu et al. proposed another expression that the dangling bonds at the grains due to Te deficiencies in polycrystalline Bi_2_Te_3_ can be seen as fractional V_Te_, which can be considered the same as whole-V_Te_ defects in the grains. The abundant fractional V_Te_ around the grain boundary can induce excessive electron, which may be another reason for the n-type polycrystalline Bi_2_Te_3_ [55]. Although there are abundant defects in both monocrystalline and polycrystalline Bi_2_Te_3_ which can induce excessive carriers, the carrier concentration of both monocrystalline and polycrystalline pristine Bi_2_Te_3_ is not in the most optimized region, which leads to a limited *ZT* value. To tune the carrier concentration to the optimized region, alloying with Sb for p-type or alloying with Se for n-type is a widely used method [22, 55, 56, 59, 60]. As mentioned earlier, the smaller difference in electronegativity is corresponding to a higher possibility to form antisite defects [50]. According to the theory, the formation energy of antisite defects can be summarized as follows [56]:

(9) $$\begin{array}{c} E_{AS}\left(\text{Sb}‐\text{Te}\right)<E_{AS}\left(\text{Bi}‐\text{Te}\right)<E_{AS}\left(\text{Sb}‐\text{Se}\right)<E_{AS}\left(\text{Bi}‐\text{Se}\right) \end{array}$$

Therefore, after alloying with Sb, because the difference in electronegativity between Sb (2.05) and Te (2.10) is smaller than that between Bi (1.9) and Te (2.10), the Sb_Te_′ antisite defects have a lower formation energy and thereby can increase the acceptor defects, increasing the hole concentration in p-type Bi_2_Te_3_-based alloys. On the other hand, the larger difference in size between cations and anions, the more possibility to form anion vacancy [61]. In this case, via alloying Se in Bi_2_Te_3_, the larger size difference between Se and Bi can facilitate the formation of V_Se_^••^ and increase the concentration of electrons. Besides, the enthalpy of vaporization and boiling point of Se (95.48 kJ/mol, 958.15 K) is much lower than that of Te (114.1 kJ/mol, 1261 K) and Bi (151 kJ/mol, 1837 K) [54], thereby Se is more readily to evaporate, contributing to higher donor concentration. In addition, according to Equation (9), the formation energy of Bi_Se_′ is the highest, which means that alloying with Se will not induce excessive acceptor defects. In combination with the above three factors, alloying Se in Bi_2_Te_3_ can increase the concentration of donor defects, thereby providing an effective method to tune the carrier concentration to the most optimized value for n-type Bi_2_Te_3_-based alloys. Figure 3(c) shows the carrier concentration at room temperature of the Bi_2_Te_3−*x*_Se_*x*_ for both single crystals and polycrystalline materials [56, 58, 62]. In terms of single crystals, pristine Bi_2_Te_3_ show a weak p-type conductivity mechanism. With the increasing content of the extrinsic dopant Se, the Bi_2_Te_3−*x*_Se_*x*_ gradually transform into an n-type conductivity mechanism, which can be attributed to the increasing concentration of V_Se_^••^ and the compensated concentration of antisite defects. As for the polycrystalline Bi_2_Te_3_, after alloying Se with 0.1~2.0 content, the carrier concentration is increased compared with pristine Bi_2_Te_3_ which can also be explained as mentioned above. However, the carrier concentration is not monotonously increasing with the increasing Se content, which reaches the minimum at the composition of Bi_2_Te_2_Se and then increases with the increasing Se content. The abnormal phenomenon might be because of the combined action between the donor-like effect and Se vacancies [56]. According to Equation (8) [58, 59], the reduced antisite defects caused by Se alloying means a decrease in the reactant, leading to a decrease in electron concentration, while the increasing concentration of V_Se_^••^ leads to the increase in electron concentration. Therefore, the carrier concentration first decreases and then increases with the increasing Se content. As shown in Figure 3(d), the *ZT* value of Bi_2_Te_3−*x*_Se_*x*_ alloys reach the maximum around ~1.0 for Bi_2_Te_2.3_Se_0.7_ prepared via ball milling and hot deformation once [56]. The achievement in Bi_2_Te_3_-based alloys highlights the point defect engineering for achieving the most optimized carrier concentration and enhanced *ZT* value.

**Figure 5:**
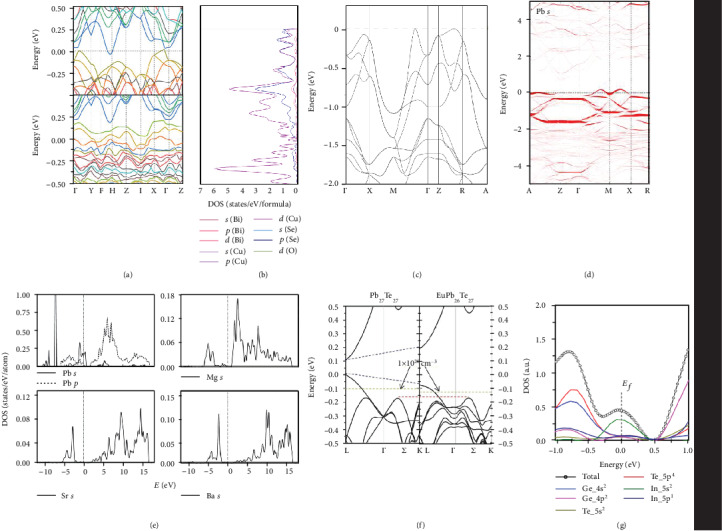
Influence on electronic band structure and DOS via inducing point defects. (a) Calculated electronic band structure of pristine Cu_2_SnSe_3_ (top) and Cu_2_SnSe_3_ with Sn vacancy (bottom) [63]. Reproduced with permission from the American Chemical Society. (b) Projected partial DOS of pristine BiCuSeO [64]. (c) Band structure of pristine BiCuSeO [64]. Reproduced with permission from the American Physical Society. (d) Orbital-resolved band structures of Bi_0.875_Pb_0.125_CuSeO; the line width reflects the weight of impurity Pb *s* states in bands [46]. (e) Orbital-projected density of states in BiCuSeO doped with Pb, Mg, Sr, and Ba [46]. Reproduced with permission from WILEY-VCH. (f) Band structure of Pb_27_Te_27_ and EuPb_26_Te_27_ [20]. Reproduced with permission from WILEY-VCH (g) DOS of Ge_0.99_In_0.01_Te with a rhomboidal phase [72]. Reproduced with permission from WILEY-VCH.

The foregoing description mainly considers the physical effects introduced by defects in real space, but from the perspective in a reciprocal space, the band structure of the material is a fundamental factor which determines the electrical operation of the material. The development of computational material science enables us to predict the band structure of materials and the effects of defects on the electronic structure, which can serve for the design of thermoelectric performance optimization. The following will focus on the effect of defects on the electronic band structure, and the structure-property relationship between electronic structure and the electronic transport behavior.  Figure 5(a) shows the Fermi level shift originating from inducing Cu vacancies in Cu_2_Sn_1−*y*_Se_3_ materials. After inducing Sn vacancies, the Fermi level is deeply sunk in the valence band, exhibiting a characteristic of a strong degenerate semiconductor. In accordance with the downshift Fermi level, the increased carrier concentration is observed and multiband around the downshift Fermi level can be involved in the electrical transportation. In Cu_2_Sn_0.93_Se_3_, *ZT* value of 0.87 is achieved at 800 K which is 67% higher than the pristine sample [63]. Except for moving the position of the Fermi level, the point defects may induce impurity levels contributing to an optimization of electrical properties. Figures 5(b) and 5(c) show the calculated density of state (DOS) and band structure of pristine BiCuSeO [64], and Figure 5(d) shows the orbital-resolved band structure of Pb-doped BiCuSeO in which the line width reflects the weight of *s* states of Pb impurity [46]. It can be observed that the Pb *s* state contributes a large proportion to the energy band around the Fermi level, which can be considered that Pb *s* state forms impurity levels exhibiting a characteristic of a strong degenerate semiconductor. Benefited from the physical effects induced via Pb doping, the carrier concentration is boosted from 6.18 × 10^18^ cm^−3^ up to ~10^20^ cm^−3^. The significant enhancement in carrier concentration leads to the increase in electrical conductivity. Besides, it is worthwhile to point out that Pb is one of the most efficient dopants for the BiCuSeO system, and the calculated DOS can give an intuitive interpretation. As shown in Figure 5(e), the DOS around the Fermi level of Mg-, Sr-, and Ba-doped BiCuSeO does not have an obvious enhancement compared with pristine composition. However, after doping with Pb, the DOS around the Fermi level gains a significant improvement, which means more carriers can be activated to the electrical transport process. The significant improvement of DOS around the Fermi level gives another explanation why Pb doping is an exceeding effective dopant for optimizing the *ZT* value in the BiCuSeO system [46]. In addition to the relative positions of impurity levels and Fermi levels, the relative positions of impurity levels and VBM or CBM also deserve our attention. Shallow level impurity states are close to the band edge, therefore easy to ionize to contribute extrinsic carriers, which are widely adapted to tune the carrier concentration in thermoelectric materials. On the contrary, deep level impurity states are separated by at least 100 meV from the VBM or CBM, therefore having a relatively large ionization energy and are hard to ionize [65]. But, when the temperature is increased, the enlarged thermal energy can promote the deep level impurities to ionization, hence realizing *T*-dependent carrier concentration regulation. Taking advantage of this feature, Su et al. achieved an enhancement in the average *ZT* value in a Ga-doped PbTe system [66].

In addition, inducing point defects to realize band convergence is also a feasible method to optimize electrical properties from the points of view in the reciprocal space. According to the simple theory for nearly free electrons, the Seebeck coefficient for metals or degenerated semiconductors can be given as [67]

(10) $$\begin{array}{c} S=\left(\pi^{2}{k_{\text{B}}}^{2}T/3e\right)\left(8{m_{d}}^{*}/h^{2}\right){\left(\pi /3n\right)}^{2/3}\left(1+r_{x}\right), \end{array}$$

where *m*_*d*_^∗^ is the effective mass of density of state, *k*_B_ is the Boltzmann constant, *h* the Plank constant, and *r*_*x*_ is the scattering parameter, from which we can find that supposed the carrier concentration *n* is constant, large *m*_*d*_^∗^ will contribute to a large Seebeck coefficient once *r*_*x*_ is settled in energy-independent scattering approximation [68]. The *m*_*d*_^∗^ can be given as [11]

(11) $$\begin{array}{c} m_{d}^{*}=N_{V}^{2/3}m_{b}^{*}, \end{array}$$

in which *N*_*V*_ is the number of degeneracy band valley and *m*_*b*_^∗^ is the band effective mass. Evidently, the enhancement in *N*_*V*_ can contribute to the enhancement in *m*_*d*_^∗^ and *S*. The feasibility of band convergence to increase *N*_*V*_ via point defect engineering has been proved in recent years [20, 69–71].  Figure 5(f) shows the calculated band structure of PbTe and Eu-doped PbTe. As for the pristine PbTe, the energy offset between the two valence bands (Δ_L−*Σ*_) is 0.16 eV. By means of substituting Pb with Eu, the Δ_L−*Σ*_ decreases to 0.08 eV, which means increasing the energy overlap of the two valence bands, and Eu doping can effectively converge the valence band. As a consequence, the converged band leads to the increase of *m*_*d*_^∗^ and thereby enlarges the Seebeck coefficient [20]. In order to increase the Seebeck coefficient, inducing dopant atoms to form a resonant level is another feasible choice. According to the Bethe-Sommerfeld expansion of Mott relation for degenerate statistics and single band conduction, an approximated formula describing the relation between the Seebeck coefficient and DOS at the Fermi level is given as follows [2]:

(12) $$\begin{array}{c} S=\frac{\pi^{2}k_{\text{B}}^{2}T}{3e}{\left.\left(\frac{DOS\left(E\right)}{n\left(E\right)}+\frac{d\mu \left(E\right)}{\mu \left(E\right)dE}\right)\right|}_{E=E_{\text{F}}}, \end{array}$$

According to Equation (12), if the influence on the carrier mobility can be neglected, the larger DOS at the Fermi level will devote to a larger Seebeck coefficient. The resonant doping means inducing dopant atoms to create resonant levels, which expressly increases the DOS around the Fermi level and thereby contribute to an enhanced Seebeck coefficient. The resonant doping has been achieved in GeTe- [72, 73], SnTe- [26], and PbTe- [74] based materials and so on.  Figure 5(g) shows the calculated partial DOS for In-doped GeTe. Apparently, the dopant In-5s energy level devotes a large DOS around the Fermi level, which causes a surge for the total density of state around the Fermi level. As a result, the Seebeck coefficient is significantly enhanced and a high *ZT* value of 2.3 is achieved for Ge_0.89_Sb_0.1_In_0.01_Te [72].

Reducing the thermal conductivity is also an important part of improving the thermoelectric properties of materials. The electronic thermal conductivity mainly depends on the manipulation on the electrical properties mentioned above, which is strongly related to electrical conductivity and Lorenz factor according the Wiedemann-Franz law [75]. While the lattice thermal conductivity is relatively independent from the electrical properties, it has a strong reliance on the phonon transport process. In bulk materials, the lattice thermal conductivity is described as [76]

(13) $$\begin{array}{c} \kappa_{L}=\frac{1}{3}\int_{0}^{\omega_{\max}}C_{S}\left(\omega \right)\;v_{g}^{2}\left(\omega \right)\;\tau_{p}\left(\omega \right)\;d\omega , \end{array}$$

where *Cs* is the spectral heat capacity, *v*_*g*_ is the group velocity, and *τ*_*p*_ is the phonon relaxation time. Because the high thermoelectric performance needs the thermal conductivity as low as possible, *τ*_*p*_ should be reduced into the minimum. Materials with high lattice anharmonicity, which intensifies the Umklapp phonon–phonon scattering and is closely related to intrinsic chemical bonding, and crystal structure, has been shown to obtain short intrinsic phonon relaxation time in PbTe and SnSe, which pushes us to search for new material systems with high lattice anharmonicity [9, 10]. While for a given material system, *τ*_*p*_ is strongly related with the defects in the materials which intensifies the phonon scattering process, and point defects, linear defects, planar defects and bulk defects are all enabled to scatter phonons. The scattering probability which originated from each type of scattering source is related to the defect concentration and has its own frequency (*ω*) dependence [77]. Here, we mainly discuss how point defects influence the *κ*_*L*_; the effects of other types of defects will be discussed in the next parts. Point defects effectively scatter phonons with high frequency in a relaxation time of *τ*_*PD*_^−1^~*ω*^4^ [77, 78]. According to Klemens' [79] and Callaway's [80, 81] theory, the introduction of point defects can cause mass contrast effect and strain field fluctuation effect, both of which devote to the reduction in *κ*_*L*_. In order to assess the degree of the reduction in *κ*_*L*_ due to the two effects, Abeles put forward the scattering parameter (Γ) which can be given as follows [82]:

(14) $$\begin{array}{c} \Gamma =x\left(1-x\right)\left[{\left(\frac{\Delta M}{M}\right)}^{2}+\varepsilon {\left(\frac{\Delta \delta }{\delta }\right)}^{2}\right], \end{array}$$

where *x* is the doping concentration, *ε* is a phenomenological and adjustable parameter, and Δ*M*/*M* and Δ*δ*/*δ* are the rates of change between the defect and host atom of atomic weight and radius, respectively. From Equation (14), we can find that by means of inducing point defects, high concentration of point defects, high difference in atom mass, and high lattice distortion will lead to low *κ*_*L*_. In this case, as for the same point defect concentration, vacancy and interstitial atoms can be inferred to have a remarkable ability to reduce *κ*_*L*_, because they have the maximal Δ*M*/*M* and Δ*δ*/*δ*, which means vacancy and interstitial can introduce the most effective mass fluctuation and strain fluctuation effect. The strategy to reduce *κ*_*L*_ via inducing vacancy and interstitial has been widely used in various material system such as BiCuSeO [83, 84], Bi_2_O_2_Se [85], SnTe [70], CuGaTe_2_ [86], and CoSb_3_ [87–91]. Li et al. intentionally induced dual cation vacancy in BiCuSeO, and found that the *κ*_*L*_ of samples with single cation vacancy is lower than the pristine sample, and the *κ*_*L*_ of the samples with both Bi^3+^ vacancy and Cu^+^ vacancy is lower than the former [84]. In SnTe-based materials, for the small atomic radius of Cu, SnTe alloyed with Cu_2_Te can produce half of Cu substitution and half of Cu interstitial, which realizes the record lowest *κ*_*L*_ and partially proves the virtue of interstitial atoms [70]. Besides, in filled skutterudite systems, the filler atoms can also be regarded as a kind of interstitial atoms, and various kinds of elements such as rare earths [87, 88], alkalines [89], and alkaline earths [90] have been applied to fill the huge lattice cages, achieving a large reduction in *κ*_*L*_. Particularly, multiple filling atoms can further reduce the *κ*_*L*_ in Ba_*x*_La_*y*_Yb_*z*_Co_4_Sb_12_ compared with single filling. The results can be explained that different kinds of filling atoms have distinct resonant frequency, and the alliance of them induces multiple mode of vibration achieving the wide spectrum phonon scattering [91]. It is worth noting that the mass contrast and strain field fluctuation model may not work when the phonon spectrum is changed a lot when the scattering effect inducing from defects cannot just be handled as a perturbation.

Although the introduction of a certain type of point defect will affect the electrical and thermal transport parameters of the material at the same time, the introduction of a single type of point defect generally cannot improve the thermoelectric performance to the most optimized value, thereby researchers often introduce multiple point defects to synergistically optimize the thermoelectric properties of materials. For instance, Li et al. simultaneously induced Cu interstitial and Mn substitution in Sn_0.86_Mn_0.14_Te(Cu_2_Te)_*x*_, in which Cu interstitial mainly reduce the lattice thermal conductivity and Mn substitution realizing the band convergence optimizing the electrical properties, contributing to a record *ZT* value of 1.6 [70]. Yu et al. induced Zr and Sb codoping in Hf_1-__*x*_Zr_*x*_NiSn_1-__*y*_Sb_*y*_ half-Heusler alloys, in which Sn substitution optimizes the carrier concentration enhancing the power factor and Zr substitution reduces the lattice thermal conductivity, and the *ZT* is optimized up to 1.0 [92]. Zhang et al. dissolved Sb_2_Te_3_ in GeTe, synchronously inducing Sb substitution and Ge vacancies for each substitution of 3 Ge^2+^ by only 2 Sb^2+^ creating 1 Ge vacancy, which optimizes the hole concentration and reduces the *κ*_*L*_, realizing a significantly improved *ZT* value compared with pristine GeTe [93]. With the growing complexity of point defect types, it has become more and more important to characterize whether the defects conceived by researchers really exist in the system, which can help us to draw a clearer conclusion. Positron annihilation spectrometry (PAS) has sensitivity with ppm level to detect defects with negative charge in bulk materials, which has been employed in recent research [84, 94–96]. Figure 4(a) shows the positron lifetime spectrum of Bi_1-__*x*_Cu_1-__*y*_SeO samples with artificially introduced V_Bi_ and V_Cu_ [84]. By means of decoding the spectrum, the researchers can obtain the derived lifetime parameters *τ*_*i*_ of the samples. Furthermore, by matching the derived lifetime parameters to the theoretically calculated lifetime parameters of a certain defect in the system, the researchers can identify the main types of defects in the material. The *τ*_*i*_ in Bi_0.975_Cu_0.975_SeO can be ascribed to bulk lifetime, large size defects, interface, V_Bi_, and V_Cu_, which provides the evidence for the existence of different kinds of defects [84]. In addition, the rapid development of electron microscope technology also facilitates a direct observation of various point defects. Figure 4(b) shows the STM image of Sb_2_Te_3_, from which we can clearly observe various point defects including vacancies and antisite defects [52]. The determination of antisite defects also helps us to analyze and design thermoelectric materials. In Bi_2_Te_3-__*x*_Se_*x*_ system, if we judge the influence of Se alloying only according to isoelectronic trap principle while without the analysis of antisite defects, the Se alloying would be p-type doping for the stronger ability to attract electrons of Se atoms, which violates the n-type experimental fact as aforementioned. The determination of antisite defects in Bi_2_Te_3_ based materials explains the abnormal sense and helps us to better design the Bi_2_Te_3_-based materials when optimizing carrier concentration and attempting other doping elements.

In addition to simple point defects mentioned above, complex point defects composed of several lattice sites such as cross-substitution, vacancy cluster, and interstitial cluster may also exist in the thermoelectric materials and have distinctive physical effects. The cross-substitution shown in Figure 1 means substitution at lattice sites adjacent to one host atom by pairs while maintaining the balance of the chemical valence. For example, in the AgPb_*m*_SbTe_2+__*m*_ system, Pb^2+^ lattice sites around one Te^2-^ site can be replaced by Ag^+^ and Sb^3+^ pairs, which still maintain the balance of chemical valence [30]. The cross-substitution has a higher concentration compared with single dopant and therefore can have a relatively strong phonon scattering effect [3]. Shi et al. induced cross-substitution in type I clathrates via substituting Ge by Ga and transition metals, which increases the ionized impurity scattering and phonon scattering, enhancing the power factor and reducing thermal conductivity, respectively [97]. Interstitial pairs, which mean double interstitial atoms simultaneously occupying the interspace in the crystal lattice, can also exist especially in the system with large interspace such as skutterudites. Figure 4(c) shows the typical skutterudite crystal structure of CoSb_3_ with single Co interstitial and Co interstitial pair complex defects, respectively. In order to provide evidence for the existence of Co interstitial pair complex defects, Li *et al.* calculated the formation energy as a function of Fermi level in Co rich and Sb rich regions, respectively, as shown in Figure 4(d). In the case of Co rich, the formation energy of Co interstitial pair (purple line) with -1 charge state is the lowest across the entire Fermi level, which implies that Co interstitial pair is the dominant point defects at low temperature. Although the Co interstitial pair has a low decomposition temperature which means Co interstitial pair may not form during the synthesis process at high temperature, the study also gives us a new sight for realizing the complex point defects in thermoelectric materials [29]. As for more complex point defects, Lee et al. puts forward that there are various possible complex point defects in Ag-doped Sn_1+*δ*_Te via formation energy calculation, including (Ag-Ag)_Sn_, (Ag_Sn_-Ag_Te_), (Ag_Sn_-Ag_Te_-Ag_Sn_)_linear_, (Ag_Sn_-Ag_Te_-Ag_Sn_)_orthogonal_, and (Ag_Sn_-Ag_Te_-Ag_Sn_-Ag_Te_)_square_. The band structures with different dominant defects show distinct difference, and the authors hold that the abnormal behavior of carrier concentration and lattice parameter variation is due to Sn vacancy and Ag complex defects [31]. Although there is no direct characterization to prove the existence of Ag complex defects, the results will undoubtedly give us an expanded horizon of complex point defects in thermoelectric materials in the future research.

Except for the types and concentration of point defects, the distribution is also an essential aspect which deserves our concern. Generally, point defects are considered randomly distributed in the host matrix. However, the arrangement of point defects can also be ordered in some specific conditions. Recently, Zunger's group has predicted that in some degenerate but gapped transparent conductors such as the Ba-Nb-O system, vacancies can be arranged in an ordered way, of which the possibility is enabled by the negative formation energies of dilute vacancies originated from the Fermi level-induced spontaneous nonstoichiometry. It is also predicted that there is a sequence of possible stable arrangement of ordered vacancies, and they exhibit different free carrier concentration and optoelectronic properties [98]. But, up to now, there are few researches studying the possible ordered arrangement of defects in thermoelectric materials and linking it with thermoelectric transport behavior. In the future research, the distribution of point defects can be an important concern which may also open up new possibilities for thermoelectric optimization.

## 3. Linear Defects

The linear defects, typically the dislocations, are one-dimensional defects around which the atoms in the crystal lattice are misaligned. On a common sense, the dislocations have a strong relationship with the mechanical properties. Numerous efforts including both experimental and theoretical calculation have been devoted to establishing the relationship [99–101]. However, the experimental research about the dependence of thermoelectric properties on dislocations is not as mature as that of the mechanical properties. In recent years, the existence of dense dislocations has been found in thermoelectric materials in several works including PbSe [19], PbTe [20], Mg_2_Si [21], Bi_2_Te_3_ [22], and Zn_4_Sb_3_ [102], which all devote to strongly reduced *κ*_*L*_.

**Figure 6:**
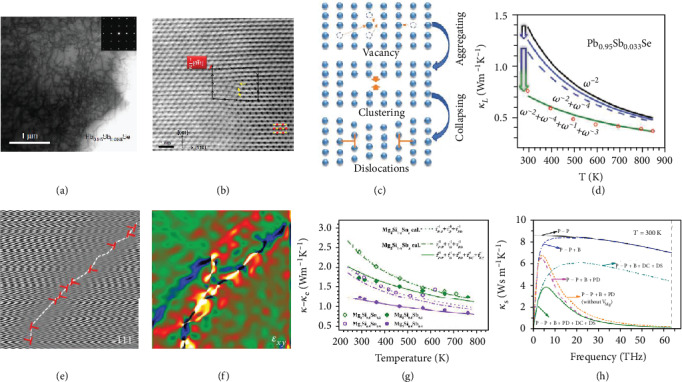
Linear defects and related physical effect in thermoelectric materials. (a) Microstructures of Pb_0.95_Sb_0.033_Se solid solution showing the uniformly distributed dense dislocation [19]. Reproduced with permission from the Nature Publishing Group. (b) High-resolution annular bright field (ABF) STEM image of Pb_0.95_Sb_0.033_Se [19]. Reproduced with permission from the Nature Publishing Group. (c) Schematic diagram of the formation of in-grain dislocations induced by vacancy clustering in Pb_1-__*x*_Sb_*x*_Se [77]. Reproduced with permission from WILEY-VCH. (d) Temperature-dependent lattice thermal conductivity for Pb_1-__*x*_Sb_2__*x*__/3_Se solid solution [19]. Reproduced with permission from the Nature Publishing Group. (e) Inverse FFT (IFFT) images of (-111) atomic plane of Sb-alloyed Mg_2_Si [21]. (f) Strain mapping of the HRTEM image along the *xy* direction in Sb-alloyed Mg_2_Si [21]. (g) Experimental data and calculated lattice thermal conductivity for Mg_2_Si_1-__*x*_Sb_*x*_ and Mg_2_Si_1-__*x*_Sn_*x*_ [21]. (h) Contribution of different phonon scattering mechanisms to the spectral thermal conductivity of the Mg_2_Si_0.8_Sb_0.2_ at room temperature [21]. Reproduced with permission from Elsevier Ltd.

Unlike point defects which can relatively easily form a high concentration in the bulk material, the dense dislocation is not familiar in thermoelectric materials, Thereby, the first concern can be how the dense dislocation forms in the thermoelectric materials. Historically, heavy plastic deformation on the bulk material has been widely used in structural steel represented by Fe-C alloys to create dislocation in order to produce a work hardening effect. However, this also places high demands on the plasticity and toughness of the bulk material, which may be not suitable for some thermoelectric materials with brittleness. In a novel manner, Chen et al. induced dense dislocation in Pb_1-__*x*_Sb_*x*_Se solid solutions via intentionally inducing cation vacancy and thermal annealing [19].  Figure 6(a) shows the uniformly distributed in-grain dense dislocation in Pb_0.95_Sb_0.033_Se, and Figure 6(b) shows the details including Burgers loop and extra half planes of atoms, both of which provide evidence for the existence of in-grain dense dislocation [19]. During the annealing process, the vacancies can diffuse and further form vacancy clusters [103]. To maintain the state with low overall energy, the closed loops of edge dislocation forms after the vacancy cluster collapsing [104]. If the concentration of vacancies is much higher than that at thermodynamic equilibrium, the climb and multiplication of dislocations will be facilitated, leading to dislocation with high density [105]. The formation mechanism of dense dislocation has been summarized in Figure 6(c) [77]. In addition to intentionally inducing vacancy, elemental doping may also facilitate the formation of dislocation, which can be seen in Na, Eu co-doped PbTe, and rare earth-doped Zn_4_Sb_3_ [20, 102]. Besides, a certain synthetic process such as liquid compaction can induce dense dislocation along the boundary. By means of inducing excessive Te in Bi_0.5_Sb_1.5_Te_3_, the excessive Te between the Bi-Sb-Te alloy grains will be expelled during the compaction process, and the dislocation will be facilitated to form around the grain boundary with low energy [22].

To rationally design and optimize the thermoelectric properties of the materials via dislocation, it is important to clarify how much dislocation is induced in the material. The modified Williamson-Hall plot has been adopted to estimate the dislocation density in PbTe, PbSe, and *β*-Zn_4_Sb_3_ [19, 20, 102]. The modified Williamson-Hall model is expressed as follows [106, 107]:

(15) $$\begin{array}{c} \Delta K≃0.9/D+{\left(\pi A^{2}b^{2}/2\right)}^{1/2}\rho^{1/2}\left(K{\bar{C}}^{1/2}\right)+\left(\pi A^{\prime }b^{2}/2\right)Q^{1/2}\left(K^{2}\bar{C}\right),\;K=2\sin\theta /\lambda , \end{array}$$

in which Δ*K* is the full-width at half-maximum (FWHM), *D* is the average crystallite size, *A* and *A*′ are constants determined by the effective outer cut-off radius of dislocations, *b* is burgers vectors, *ρ* is density of dislocation, $\bar{C}$ is the average dislocation contrast factor, *Q* is related to the two-particle correlations in the dislocation ensemble, *θ* is the diffraction angle, and *λ* is the wavelength of X-ray [106, 107]. The model establishes a strong relationship between dislocation density and parameters determined by X-ray diffraction, which means we can employ X-ray diffraction; the most common characterization method to analyse the concentration of dislocation we induced in the materials. In the Na*_y_*Eu_0.03_Pb_0.97−*y*_Te system, the dopant concentration of Na enables a transition of the major microstructural defect from point defects, to dislocations, and finally to nanoprecipitates with the increasing Na doping concentration. The dislocation density is calculated via the Williamson-Hall model, and the results show that the density of dislocation reaches the maximum at *y* = 0.025 and then decreases with increasing *y*, which qualitatively supports the conclusion of the transition of defects [20]. The Williamson-Hall model also has been used in rare earth-doped *β*-Zn_4_Sb_3_. The rare earth dopant atoms induce excessive nonintrinsic Zn vacancies, which motivates the migration of vacancies and generates the dislocations. Meanwhile, the rare earth dopants act as the multiplication sites for dislocations and Bardeen-Herring source, which helps the dislocations climb through the migration of Zn vacancies. Here, the Williamson-Hall model is employed and the result shows that the slopes of rare earth-doped *β*-Zn_4_Sb_3_ in the Williamson-Hall plot are enhanced compared with the pristine *β*-Zn_4_Sb_3_, which provides an evidence that the introduction of rare earth dopant facilitates the formation of dislocations in *β*-Zn_4_Sb_3_ [102]. However, except for the dislocation density, elemental doping and grain size related to boundary density can influence the FWHM as well, but these works in which extrinsic doping atoms are induced do not take enough attention on the influence of doping atoms, which is recommended to take more consideration in future research when employing the modified Williamson-Hall plot. In addition, it is worthwhile to stress that the service temperature regions of many thermoelectric material systems are in the medium-temperature region or the high-temperature region, in which the recovery of dislocations may occur and thus reduce the dislocation density [108]. Hence, the thermal stability of dislocations in the thermoelectric materials should be another essential concern when inducing dense dislocations in thermoelectrics in the future research.

As for the influence on electrical properties, the dislocation can also produce carrier scattering effect, and Pödör put forward a model describing the dislocation-induced carrier scatter mechanism [109]. Taking an n-type semiconductor for example, the dislocation with edge component can introduce acceptor centers along the dislocation line capturing electrons from the conduction band [109, 110]. The dislocation lines become negatively charged, and the potential field is established around the dislocation line, which can scatter electrons and decrease the carrier mobility. According to the model, the temperature dependence on carrier mobility can be described as follows [109]:

(16) $$\begin{array}{c} \mu_{\text{c},\text{disl}}=\frac{30\sqrt{2}\varepsilon^{2}a^{2}{\left(kT\right)}^{3/2}}{N_{D}e^{3}{f_{a}}^{2}\lambda_{D}m^{1/2}}, \end{array}$$

where *N*_*D*_ is the concentration of dislocation, *λ*_*d*_ is the Debye screening length, *f*_*a*_ is the occupation rate of the acceptor centers, from which we can find high concentration of dislocation will be a harm to carrier mobility and the relationship *μ*_*c*,*disl*_ ∝ *T*^3/2^ can be established. The availability of the model can be suitable for GaN and Ge at a low temperature [109, 111]. However, temperature dependence is not observed in Pb_1-__*x*_Sb_2__*x*__/3_Se with dense dislocation, which is possibly because the dislocation-induced carrier scattering is screened due to the high dielectric constant of PbSe [19].

For thermal properties, dislocations strongly scatter phonons with middle frequency [77, 78, 112].  Figure 6(d) shows the experimental and calculated *κ*_*L*_ in consideration of different scattering mechanisms [19]. Compared with the calculated *κ*_*L*_ in consideration only with Umklapp scattering and point defect scattering (blue dashed line), the *κ*_*L*_ with an additional consideration of dislocation is heavily reduced, which shows the feasibility to reduce *κ*_*L*_ via inducing dislocations. Phonon scattering by dislocation mainly originated from two aspects: one of which is the atomic misalignment at core of the dislocations and the second is the strain field around the dislocation. The reciprocal phonon relaxation time originated from the scattering of core of the dislocation (*τ*_DC_^−1^) and strain (*τ*_DS_^−1^) can be described as follows [113, 114]:

(17) $$\begin{array}{c} \tau_{DC}^{-1}=N_{D}\frac{{V_{0}}^{4/3}}{v_{\text{s}}^{2}}\omega^{3}, \end{array}$$

(18) $$\begin{array}{c} \tau_{\text{DS}}^{-1}=0.6B_{D}^{2}N_{D}\gamma^{2}\omega \left\{\frac{1}{2}+\frac{1}{24}{\left(\frac{1-2v_{p}}{1-v_{p}}\right)}^{2}{\left[1+\sqrt{2}{\left(\frac{v_{l}}{v_{t}}\right)}^{2}\right]}^{2}\right\}, \end{array}$$

where *N*_*D*_ is the density of dislocations, *v*_s_ is the average speed of sound, *ω* is the average phonon frequency, *B*_*D*_ is the effective Burger's vector, *V*_0_ is the volume of the primitive cell, *γ* is the Gruneisen parameter, *v*_*p*_ is the Poisson ratio, *v*_*l*_ is the longitudinal phonon velocity, and *v*_*t*_ is the transverse phonon velocity. According to Equations (17) and (18), the larger effective Burger's vector and density of dislocation will cause a larger dislocation-induced phonon scattering effect.  Figure 6(e) shows the inverse FFT (IFFT) images of (-111) atomic plane of Sb-alloyed Mg_2_Si, which provides the evidence for the existence of dense dislocation [21].  Figure 6(f) shows the strain mapping along *xy* direction, and asymmetric strain field originated from the dislocation can be inferred to cause strong phonon scattering [21].  Figure 6(g) shows the calculated lattice thermal conductivity and experimental data of the Mg_2_Si_1-__*x*_Sb_*x*_ and Mg_2_Si_1-__*x*_Sn_*x*_. As for Mg_2_Si_1-__*x*_Sn_*x*_, the experimental data are in good agreement with the theoretical calculated data in consideration of phonon-phonon scattering, boundary scattering, and point defect scattering. However, the obvious deviation between the experimental and calculated data is found in Mg_2_Si_1-__*x*_Sb_*x*_, according to which other scattering can be inferred to exist. In consideration with dislocation-induced scattering and engaging with Equations (17) and (18), the calculated data (solid line) have a good agreement with the experimental data, which indicates that the dense dislocation can strongly scatter the phonon and further reduce the lattice thermal conductivity.  Figure 6(h) is the calculated spectral thermal conductivity of the Mg_2_Si_0.8_Sb_0.2_ indicating the contribution of different phonon scattering mechanisms. From the image, we can find that the point defects mainly scatter the phonon with high frequency, while the dislocation scatters both phonon with middle frequency and high frequency, and the *κ*_*s*_ in consideration of both point defects and dislocation is the lowest. Combined with inducing point defects, the *κ_L_* of Sb-doped Mg_2_Si with dislocations is significantly reduced and lower than that of Sn-doped Mg_2_Si without dislocation [21].

## 4. Planar Defects

**Figure 7:**
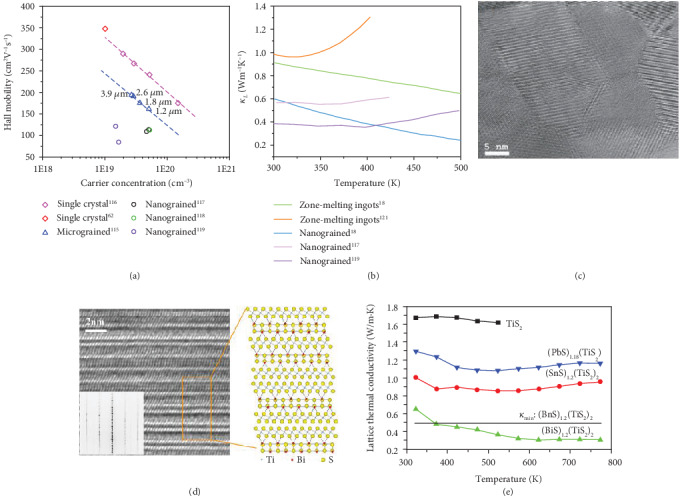
Planar defects and related physical effect in thermoelectric materials. (a) Hall mobility and carrier concentration of single crystals and polycrystalline materials with different grain sizes in Bi_2_Te_3_-based alloys. Data are from Ref. [62, 115–119]. (b) Temperature-dependent lattice thermal conductivity of Bi_2_Te_3_-based alloys. Data are from Ref. [18, 117, 119, 121]. (c) TEM images showing the clean grain boundaries of Bi_0.5_Sb_1.5_Te_3_ nanograins [18]. Reproduced with permission from the American Association for the Advancement of Science. (d) TEM image, SAED image, and simulated crystal structure of (BiS)_1.2_(TiS_2_)_2_ showing the stack faults [129]. (e) Temperature-dependent lattice thermal conductivity of TiS_2_ and (MS)_1+__*x*_(TiS_2_)_2_ (M = Pb, Sn, and Bi) [129]. Reproduced with permission from AIP Publishing.

The planar defects are two-dimensional defects where the lattice deviates from the periodic lattice structure with a large size along the in-plane direction while with a very small size along the cross-plane direction, such as stacking faults, grain boundaries, and twin boundary, among which grain boundaries are the most common planar defects in thermoelectric materials. Benefited from the development of material processing technique, the size of the grains can be successfully reduced to the micrometer or even the nanometer level, drawing a new picture of size-related physical effects deviated from conventional materials. The gradual reduction in grain size is also accompanied by the increase in grain boundary density, which is widely distributed in polycrystalline materials. Hence, it is necessary to have an in-depth comprehension of the effect of grain boundaries on the thermoelectric property. As for electrical transport properties, on the one hand, the disorder engaged with more atoms can build up an interfacial potential deviated from the periodic potential field, which scatters the carrier and deteriorates the carrier mobility. On the other hand, around grain boundaries it's always enriched with various other types of defects such as dangling bond, point defects, and precipitates influenced from the interfacial potential due to periodic potential field interruption. Kim et al. systemically studied the effect of grain boundary in Bi_2_Te_3_. By controlling the soaking time during the hot-press process, the grain size is reduced with the limiting soaking time. As shown in Figure 7(a), the carrier mobility of polycrystalline is reduced compared with single crystal, and the carrier mobility is also decreased with the increasing density of grain boundaries [62, 115, 116]. The phenomenon can be explained from the enlarged scattering effect which originated from the increasing density of grain boundaries. When the grain size is further reduced into nanoscale, further reduction in the carrier mobility can be observed, which is consistent with the enhanced carrier scattering effect caused by denser grain boundaries [117–119].

As for the thermal transport properties, grain boundary is generally the dominant scattering source for low-frequency phonons in a relaxation time of *τ*_*B*_^−1^~*ω*^0^  [77, 120]. Besides, as low-frequency phonon dominates the heat-transporting process at low temperature, the boundary-induced phonon scattering effect can be expected to strongly reduce the lattice thermal conductivity at low temperature [77]. With the development of material processing technique such as high-energy ball milling, spark plasma sintering (SPS), and melting-spin, the grain size is enabled to be reduced into nanometer level which breaks through the traditional limitation in the reduction of thermal conductivity. During the synthesis process, ball milling enables the nanoscale of fine powders, and the SPS processing can prevent excessive grain growth during compaction. Figure 7(c) shows the clean grain boundaries of Bi_0.5_Sb_1.5_Te_3_ in the nanometer scale prepared via ball milling [18]. The dense grain boundaries intensify the phonon scattering process and finally enable the reduction in lattice thermal conductivity. As shown in Figure 7(b), the reduction in lattice thermal conductivity of Bi_2_Te_3_-based alloys can be obviously observed when the grain size is narrowed into nanoscale, indicating the virtue of nanostructured materials [18, 117, 119, 121]. Besides, Poudelthe et al. put forward that in the Bi_0.5_Sb_1.5_Te_3_ system, the bipolar conduction is compensated at high temperature, which can be attributed that dense boundaries create an interfacial potential which scatters more electrons than holes. In addition, the dense boundaries facilitate antisite defects to accumulate around them, which contribute to the increase in electrical conductivity. Profited from the nanosized grains, a *ZT* value of 1.4 at 373 K is achieved in p-type Bi_0.5_Sb_1.5_Te_3_ alloys [16]. The optimization strategy of reducing the grain size has also been achieved in PbTe [122], SiGe [123, 124], and CoSb_3_ [125]. In addition to the reduction of the grain size, the introduction of the second phase in nanoscale can also cause boundary-related effects, and this part will be discussed in detail in the next section.

Stacking faults are generally categorized as planar defects which mean a local deviation from the regular stacking order, such as the “AABBAA” stacking order occurring in crystals with “AABAA” intrinsic stacking order [101, 126]. The disorder among atomic layers can also be the source of phonon scattering and reduces the lattice thermal conductivity. For instance, (MS)_1+__*x*_(TiS_2_)_2_ is a kind of thermoelectric materials with natural superlattice, in which TiS_2_ layers provide favorable electrical properties, and intercalated MS layers causes the phonon scattering effect to reduce lattice thermal conductivity [127–129]. Wan et al. found that the stacking default in (BiS)_1.2_(TiS_2_)_2_ can systematically reduce the *κ*_*L*_ to an exceedingly low level. As shown in Figure 7(d), for most of the area, double TiS_2_ layers and single BiS layer stack alternatively. However, in some areas, single TiS_2_ layers and single BiS layer stack alternatively, indicating a stacking default in the bulk material. The SAED image in Figure 7(e) shows that reflection spots are directionally elongated, which also indicates the noticeable disorder along the stacking direction. As a consequence, shown in Figure 7(e), the lattice thermal conductivity is strongly reduced to around 0.3 W/m K which is remarkably ultralow among thermoelectric materials. Besides, (BiS)_1.2_(TiS_2_)_2_ has a lower lattice thermal conductivity compared with TiS_2_, (PbS)_1.18_(TiS_2_)_2_, and (SnS)_1.2_(TiS_2_)_2_ with little disorder across the planes which is supported by SAED [129]. The results reveal that stacking default can cause strong phonon scattering effect and effectively reduce the lattice thermal conductivity, which provides new ideas for thermoelectric performance optimization.

## 5. Bulk Defects

The bulk defects are three-dimensional defects with a relative large scale which diffusely distribute in the matrix, including the guest phase, pores, and cracks. The guest phase can be caused by an incomplete chemical reaction and the precipitation from the matrix during the cooling process. And it may also be triggered on purpose by the researchers. When the guest phase particles are added, on the one hand, the guest phase causes interface-related effects. The guest phase can form a localized heterojunction with the matrix for semiconductor materials, and the potential field will be formed at the interface and thus generate scattering effect on the carrier transport process. At the same time, the introduction of new grain boundaries will strongly scatter phonons with low frequency, thereby reducing the lattice thermal conductivity of the system. On the other hand, the transport characteristics of the guest phase particles themselves will also influence the transport performance of the system.

**Figure 8:**
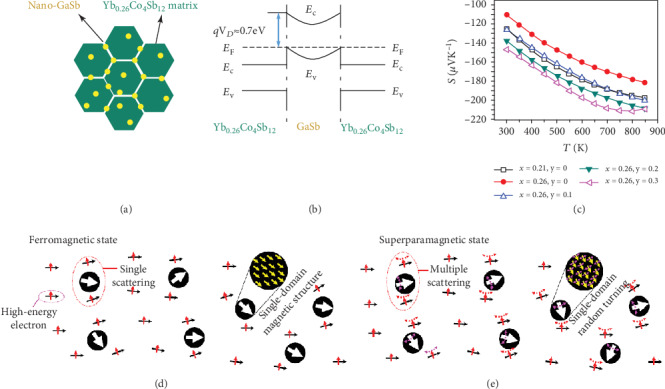
Bulk defects and related physical effects in thermoelectric materials. (a–c) Introduction of energy filtering effect in Yb_0.26_Co_4_Sb_12_/nano-GaSb composites, which increases the Seebeck coefficient [25]. Reproduced with permission from Elsevier Ltd. (d, e) Introduction of the influence of magnetic inclusions on carrier transport behavior in the (d) ferromagnetic state and (e) superparamagnetic state, respectively [132]. Reproduced with permission from Springer Nature.

The energy filtering effect is an energy-dependent carrier scattering effect which originated from the inducement of the second phase, which forms an additional energy barrier at the interface [130]. Figures 8(a)–8(c) introduce the energy filtering effect in Yb_0.26_Co_4_Sb_12_/nano-GaSb composite materials. The host phase Yb_0.26_Co_4_Sb_12_ is an n-type conduction, while the guest phase nano-GaSb is p-type conduction, and they have almost the same Fermi level based on the work function measurement. When the matrix is in contact with inclusions, an additional barrier is formed at the interface due to the relatively high-energy conduction band of GaSb. For matrix CoSb_3_ where electron transport predominates, low-energy electrons cannot cross the additional barriers and thereby are scattered, while high-energy electrons can cross the energy barrier and contribute to electrical transportation, thereby increasing the average energy of the electrons involved in transport and Seebeck coefficient which depends on the mean carrier energy near the Fermi level [25]. Energy filtering has also successfully been utilized in n-type PbTe/Ag, n-type PbTe/Pb, n-type Co_4_Sb_12_/Co, and organic and inorganic PANI/SWNT/Te composite [130–133].

In addition to the interface effects introduced by the second phase, the properties of inclusions themselves will also affect the electrical properties of the material. For instance, for the material with intrinsic low electrical conductivity, the addition of the second phase with high electrical conductivity can be a feasible choice. In this case, the percolation phenomenon is noticeable, which means the electrical conductivity increase sharply when the content of the second phase reaches the percolation threshold (*f*c) [134]. The strategy has been applied in BiCuSeO with low intrinsic electrical conductivity and Cu_2_Se with moderate conductivity composite, and a sharp increase in carrier concentration is realized when the concentration of Cu_2_Se above the percolation threshold which contributes to the enhancement in *ZT* value [135].

What is even more interesting, the magnetic properties of the second phase may also have a strong impact on the thermoelectric properties. Zhao et al. found that distributing nanoparticles with soft magnetism in the thermoelectric matrix can synchronously control the phonon and electron transport behaviors [132]. The superparamagnetic Co nanoparticles lead to three kinds of thermoelectromagnetic effects in Ba_0.3_In_0.3_Co_4_Sb_12_ matrix. Firstly, the Ohmic contact forms at the interface between the metallic Co nanoparticles and the matrix with n-type semiconductor properties, causing band bending and the formation of interface potential, which further causes the energy filtering effect mentioned above and thereby selectively scatters electrons with low energy. Secondly, the superparamagetic Co nanoparticles enable multiple electrons scattering. As shown in Figure 8(d), as for Co nanoparticles in the ferromagnetic state, the magnetic moment is rigid and is not affected by the spin of the high-energy conduction electrons which only enables single electron scattering. However, as for Co nanoparticles in the superparamagnetic state shown in Figure 8(e), the magnetic moment is no longer rigid and is turned randomly, enabling multiple scattering similar to the Kondo effect in which antiferromagnetic coupling enables multiple scattering. The enhanced multiple scattering increases the scattering parameter (*r*_*x*_), which contributes to the increased Seebeck coefficient according to Equation (10) [132]. Hence, the addition of superparamagnetic Co nanoparticles not only induces excessive electrons but also increases the scattering parameter due to multiple scattering, which realizes a decoupling optimization in both electrical conductivity and Seebeck coefficient. Thirdly, the nanoparticles and dense interface intensify the phonon scattering and reduce lattice thermal conductivity. Combined with the thermoelectromagnetic effects which originated from superparamagetic Co nanoparticles, a *ZT* value of 1.8 at 850 K is achieved which is 32% higher than the matrix [132]. In another relevant work, permanent magnet BaFe_12_O_19_ nanoparticles serve as an electron repository in the matrix Ba_0.3_In_0.3_Co_4_Sb_12_. Below the Curie temperature, the magnetic nanoparticles stay in the ferromagnetic state and trap electrons. While above the Curie temperature, the magnetic nanoparticles transform into the paramagnetic state and release the trapped electrons, which realizes a sharp increase of carrier concentration around the Curie temperature. Besides, the magnetic particles produce two magnetoelectronic effects including electron spiral motion and magnon-drag thermopower, and the magnetic transition of permanent nanoparticles is demonstrated to reduce the deterioration of thermoelectric properties in intrinsic excitation region [28].

The above strategies are based on the condition that the second phases are not dissolved in the matrix, but for some systems, the second phase impurities may gradually dissolve in the matrix with increasing temperature. Utilizing this phenomenon, *T*-dependent doping is employed to enhance the average *ZT* value among the service temperature. Taking the Na-doped PbTe system with Na-rich precipitates for instance, with the increasing temperature, the gradually dissolved inclusions provide excessive impurity-induced holes, which can compensate the bipolar conduction induced from intrinsic activation increasing the average *ZT* value. The strategy has been successfully utilized in PbTe/Ag_2_Te- and Na-doped PbTe composites [23, 24, 136].

Pores are another kind of bulk defects which can tune the electrical transport properties, despite inducing no excessive charges. In general, we always pursue high-density materials even up to around theoretical density for achieving higher thermoelectric performance. However, He et al. found an abnormal phenomenon that Co_1-__*x*_Ni_*x*_Sb_3_ with distributed pores around 1 *μ*m to 1.5 *μ*m shows a relatively high thermoelectric performance. The pores remarkably increase the Seebeck coefficient which can be attributed to the charge deposition at the pores, slightly decreasing the thermal conductivity but almost not changing the electrical conductivity, which proves the feasibility of inducing pores to enhance thermoelectric properties in CoSb_3_ [137]. The strategy has also been successfully utilized in the SnTe_1-__*x*_Se system [138]. However, the strategy of the introduction of pores is counterproductive in nanostructured SiGe alloys. Lee et al. put forward that the introduction of pores excessively deteriorates the electrical conductivity, even though the pores increase the Seebeck coefficient and reduce thermal conductivity, causing the reduction in the *ZT* value. Besides, the authors put forward that despite introducing pores not being feasible for SiGe alloys, it may be suitable for materials with a smaller effective mass and low relaxation time of carriers compared with SiGe alloys [139]. Thereby, although the introduction of pores is not a universal strategy, it can also be a candidate for optimizing thermoelectric properties in certain systems.

**Figure 9:**
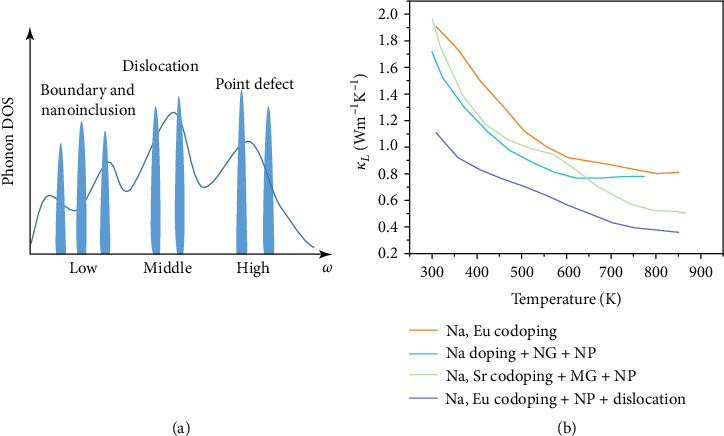
Introduction of reducing lattice thermal conductivity via inducing defects with multiple dimensions. (a) Schematic diagram of full-frequency phonon scattering that originated from frequency-dependent phonon scattering characteristics of defects with different dimensions. (b) Temperature dependence of lattice thermal conductivity in PbTe with different defects (NG: nanograins; MG: mesograins; NP: nanoprecipitates), which shows the feasibility of inducing multiple dimensional defects to reduce lattice thermal conductivity. Data are from Ref. [20, 145, 146].

As for the influence on thermal properties via inducing bulk defect, particularly for nanoparticles, the increased interface density intensifies phonon scattering for low-frequency phonons [78]. The strategy of inducing bulk defects to reduce thermal conductivity has been widely utilized in SiGe [140–142], Half-Heuslers alloys [143], CoSb_3_ [25, 132, 144], and PbTe [145, 146]. Particularly, for the materials with distributed amorphous regions, the thermal conductivity can be reduced to an ultralow level [147]. However, since the scattering of phonons by each kind of defect is limited to a specific phonon frequency, the lattice thermal conductivity of the material cannot be suppressed to the minimum by a single kind of defect. In order to further suppress the lattice thermal conductivity, defects with different dimensions can be simultaneously introduced into the material to achieve full-spectrum phonon scattering utilizing the phonon frequency-dependent scattering characteristic as shown in Figure 9(a). It is worthy to point out that, to reduce the thermal conductivity even further, defect scattering phonon with certain frequency range can be further diversified. For instance, manifold point defects can be simultaneously induced to fully scatter phonon with high frequency, and grains in mesoscale and precipitates in nanoscale can be simultaneously induced to fully scatter phonon with low frequency. Figure 9(b) shows the temperature dependence of lattice thermal conductivity in PbTe with different defects [20, 145, 146]. The Na, Eu codoping PbTe with nanoprecipitates, and dense dislocations show the lowest lattice thermal conductivity, and a rational explanation can be deduced from the realization of full-spectrum phonon scattering, among which multiple point defects scatter phonon with high frequency, dense dislocations scatter phonon with middle frequency, and nanoprecipitates scatter phonon with low frequency, realizing the maximal reduction in phonon relaxation time [20]. The experiments undoubtedly prove the dependability of the strategy of inducing multiple defects to reduce lattice thermal conductivity, which sheds light on the optimization of thermoelectric performance.

## 6. Summary and Outlook

Focused on defects in thermoelectric materials, we summarize the possible physical effects when inducing defects with different dimensions. Based on the defect-related physical effects, strategies for optimizing thermoelectric performance are extracted from the point of view in the dimension of defects. Because the introduction of defects will inevitably change nearly all the physical parameters influencing thermoelectric transport behaviors, in-depth understandings of defect-related effects with different dimensions are necessary to guide rationally manipulating defects in the materials for the optimized thermoelectric performance.

Most intuitively, defects mean the interruption of periodic potential fields and build up additional potential field, which serve as the carrier scattering sources and reduce carrier mobility. However, due to the coexistence of multiple scattering mechanisms, for a certain system abnormal phenomenon that doping increases the carrier mobility can be observed because the doping atoms change the scatter mechanism from ionized impurity scattering to mixed scattering [40, 41]. Dislocations usually serve a similar scatter mechanism compared with ionized impurity scattering. Nevertheless, due to coexistence of multiple scatter mechanisms, the characteristic is screened in certain thermoelectric systems [109, 110]. The second phases constitute localized additional potential field at the interfaces, and in a specific condition, energy filtering effects can be achieved which selectively scatter low energy carriers and enhance the Seebeck coefficient [25, 130, 131]. Besides, the magnetic nanoinclusions cause the magnetic fluctuations and a multiple scattering can be activated when the inclusions are in the superparamagnetic state which can devote to the enhancement in the Seebeck coefficient [132].

To maintain the balance in charges, the introduction of defects also tunes the carrier concentration, even for isoelectron doping, the carrier concentration can be also changed which originated from the isoelectron trap effect or changes in dominance of different defects. Inclusions change carrier concentration depends on the relative position of band structure between the matrix and inclusions. Particularly, with the rational design of the bulk defects, decoupling enhancement in both electrical conductivity and Seebeck coefficient can be achieved beneficial from the increased carrier concentration and energy filtering effect [25, 132].

From the perspective of the reciprocal space, the changes in symmetry originated from the inducement of defects reflect on the changes in the band structure, causing the Fermi level shift or sometimes realizing the band convergence contributing to enhancement in the Seebeck coefficient. Besides, the doping atoms can form impurity levels, contributing to the excitation of excessive carriers. Particularly, when the defects induce a sharp increase in DOS around the Fermi level, the resonant level can be achieved by devoting to the enhancement in the Seebeck coefficient.

As for the thermal transport behavior, the strong frequency dependence of phonon scattering by defects with different dimensions reveals the limitation of reducing *κ*_*L*_ via a single kind of defects. But, meanwhile, it also highlights the strategy to reduce *κ*_*L*_ through inducing defects with multiple dimensions to realize the full-frequency phonon scattering. In this way, *κ*_*L*_ has been reduced to an ultra-lowlevel [19–21, 146] and it also lights a feasible method for optimizing the thermoelectric performance.

Based on the current experimental achievement, it is worthwhile to note that materials designed with 1D~3D defects often intentionally combined with 0D point defects [18–22, 25, 26, 132]. The divergence between the mean free paths of phonons and electrons enable the reduction in *κ*_*L*_ via inducing planar and bulk defects while not excessively deteriorating the electrical properties, which has been the prerequisite of the strategy of construct nanostructured materials [13, 15, 17, 18]. Integrating the sensitivity to electrical properties via inducing point defects and availability of reducing *κ*_*L*_ via inducing other kinds of defects, the integrated strategy can be widely utilized in the future research aming to enhance the thermoelectric performance.

By means of integrating physical effects which originated from multiple dimensional defects induced in the materials, significant progress has been achieved in thermoelectric materials, and it also opens up a new space for optimization of thermoelectric performance in the future research. Nevertheless, it is worthwhile to note that the research on the relationship between thermoelectric performance and defects is still in its infancy. The complex point defects have been predicted to exist in the thermoelectric materials which have an impact on the band structure, but, up to now, there is no characterization to prove the direct existence of the complex point defects in thermoelectric materials. Besides, based on existing theoretical models, we can only qualitatively predict the influence on certain parameters when inducing different types of defects, but the accurate quantitative prediction of the *ZT* value which integrates electrical and thermal parameters when inducing different types of defects has not been well achieved. Facing the future demands, numerous efforts still need to be devoted to the research of thermoelectric materials on both theoretical and experimental aspects for the expanded applications of thermoelectric technology.
